# Role of Tryptophan Side Chain Dynamics on the Trp-Cage Mini-Protein Folding Studied by Molecular Dynamics Simulations

**DOI:** 10.1371/journal.pone.0088383

**Published:** 2014-02-07

**Authors:** Srinivasaraghavan Kannan, Martin Zacharias

**Affiliations:** 1 Bioinformatics Institute, Agency for Science Technology and Research, Singapore, Singapore; 2 Experimental Therapeutics Centre, Agency for Science Technology and Research, Singapore, Singapore; 3 Physik-Department T38, Technische Universität München, Garching, Germany; Consiglio Nazionale delle Ricerche, Italy

## Abstract

The 20 residue Trp-cage mini-protein is one of smallest proteins that adopt a stable folded structure containing also well-defined secondary structure elements. The hydrophobic core is arranged around a single central Trp residue. Despite several experimental and simulation studies the detailed folding mechanism of the Trp-cage protein is still not completely understood. Starting from fully extended as well as from partially folded Trp-cage structures a series of molecular dynamics simulations in explicit solvent and using four different force fields was performed. All simulations resulted in rapid collapse of the protein to on average relatively compact states. The simulations indicate a significant dependence of the speed of folding to near-native states on the side chain rotamer state of the central Trp residue. Whereas the majority of intermediate start structures with the central Trp side chain in a near-native rotameric state folded successfully within less than 100 ns only a fraction of start structures reached near-native folded states with an initially non-native Trp side chain rotamer state. Weak restraining of the Trp side chain dihedral angles to the state in the folded protein resulted in significant acceleration of the folding both starting from fully extended or intermediate conformations. The results indicate that the side chain conformation of the central Trp residue can create a significant barrier for controlling transitions to a near native folded structure. Similar mechanisms might be of importance for the folding of other protein structures.

## Introduction

**Figure 1:**
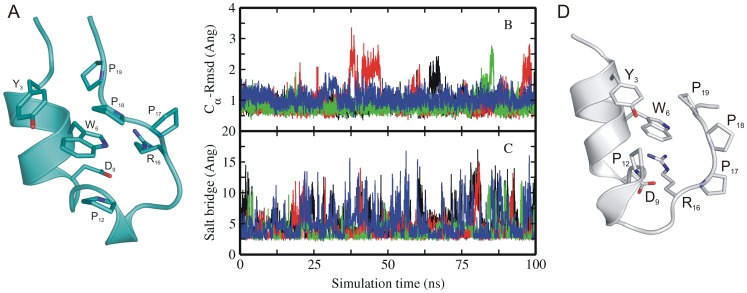
Experimental structure and MD simulations of native Trp-cage miniprotein. (A) Cartoon representation of the folded experimental structure of Trp-cage mini protein [1]. A subset of side chains important for folding are labeled (stick representation). (B) Root-mean –square deviation (RMSD) of C_α_–atoms with respect to native structure and (C) distance between Asp9-Arg16 (salt bridge) of sampled Trp-cage conformations in explicit solvent starting from the native structure (1^st^ entry of pdb 1L2Y) versus simulation time. Simulations were performed with four different force fields encoded as different line colors (black:ff03, red:ff99SB, green:ff99SB_ILDN,blue:ff99SB_NMR). (D) Partially unfolded Trp-cage structures sampled during simulation starting from native state (with the C-terminal PolyPro_II_ motive transiently dissociated from the central Trp6 residue).

Understanding the molecular details of the structure formation process of biomolecules is still a challenge in molecular biophysics and in structural biology. The Trp-cage protein is one of the smallest model proteins of just 20 amino acid residues that adopt a well defined fold with secondary structure elements and a hydrophobic core formed around a central Trp6 residue [1]. It can fold spontaneously into a stable 3D structure within ∼4 µs [2]. Structures of this protein were determined by NMR spectroscopy [1] and recently also by X-ray crystallography [3]. The Trp-cage structure contains an N-terminal α-helix, followed by a short 3_10_-helix and a C-terminal Poly-Pro_II_ (PP_II_) helix. The central buried Trp6 residue is surrounded by residues Tyr3, Leu7, Pro12, Pro18 and Pro19 (Figure 1A). The folded structure is further stabilized by a salt bridge between Aps9– Arg16. The folding mechanism of the Trp-cage protein has been extensively investigated by various experimental [2], [4]–[15] and computational [16]–[37] methods and different folding mechanism has been proposed. A consensus folding mechanism of the Trp-cage mini protein has not been obtained yet. A two stage folding mechanism was initially suggested by Qui et al [2] based on laser temperature jump spectroscopy and further supported by a thermodynamic study by Streicher et al [4] using differential scanning calorimetry (DSC) and circular dichroism spectroscopy (CD). However, other experimental studies suggest that the folding of Trp-cage mini protein does not follow a simple two state model, rather follows a more complex process involving at least one stable intermediate along the folding pathway. Neuweiler et al [5] proposed the formation of a molten globule-like intermediate along the folding pathway based on fluorescence correlation spectroscopy experiments. Using UV-resonance Raman Spectroscopy Ahmed at al [6] indicated also a more complicated folding mechanism through an intermediate molten globule state and provided evidence for α-helical structure even in the denatured state of the Trp-cage protein. Extensive hydrophobic contacts were found even in the unfolded state of the protein by Mok et al [7] by employing photochemically induced dynamic nuclear polarization (CINDP)-NMR pulse-label-experiments. In addition, recent experimental studies using NMR and Infrared (IR) T-Jump experiments [8] showed that Trp-cage folding indeed involves intermediates during the pathway where specific native and non-native contacts already pre-exist in a hydrophobically collapsed unfolded ensemble and also suggest that the N-terminal α-helix is formed already at the intermediate stage. The latter view is also supported by a recent combined T-jump/IR-spectroscopy and Molecular Dynamics (MD) simulation study [9] that indicated an intermediate state with a preformed N-terminal α-helical structure but an orientation of the C-terminal PP_II_ helix that differs from the native geometry.

Conventional MD simulations [16]–[20], [24], [28], [35] and advanced sampling simulations such as temperature replica-exchange MD simulation (T-REMD) [21], [23], [25]–[27], [34] have already been used successfully in folding simulation studies of Trp-cage using both implicit and also explicit solvation models. Even more sophisticated techniques like Hamiltonian-replica exchange (H-REMD) [22], [29]–[31] and transition path sampling [32], [33] methods have been used in theoretical folding studies. This also includes metadynamics inspired advanced sampling techniques to cover multi-dimensional energy landscapes [36], [37]. Starting from either unfolded/folded or fully extended structures, simulations were carried out for tenths to hundreds of nanoseconds and even some of them were extended to microseconds [34], [35] and successfully reached near native states with a root mean square deviation (RMSD_Cα_) of the backbone as low as ∼1 Å. Reversible folding and unfolding was observed in simulation studies based on a coarse-grained (CG) representation of the protein and surrounding CG water [38]. Reversible folding and unfolding was also achieved in atomistic explicit solvent MD simulations extending to several hundred µs close to the melting temperature of the protein [35]. Several folding mechanisms including for instance early hydrophobic collapse that is followed by either a partially folded intermediate state or just a compact denatured intermediate state stabilized by long range hydrophobic contacts with early formation of salt bridge [23] and the concurrent formation of α-helix and the hydrophobic core have been suggested [26]–[30]. Recent analysis of long MD simulations and advanced sampling simulations of the Trp-cage protein using Markov-state-models allowed the detailed characterization of the folding kinetics [39]. This simulation study identified a dominant pathway in which the N-terminal α-helix formed before or during the hydrophobic collapse in agreement with results from T-jump experiments coupled with IR spectroscopy [9].

Most simulation studies so far have focused on the pathway of folding, the variety of intermediate states, the topology and secondary structure of sampled backbone conformations and occurrence of native contacts along the folding process. In the present work all atom explicit solvent simulations have been employed to investigate the role of key contacts, in particular of the central Trp6 residue on the Trp-cage folding mechanism. Multiple classical MD simulations starting from either fully extended or various collapsed Trp-cage conformations using four different force fields have been performed. The collapsed non-native starting states showed various degrees of similarity in secondary structure and side chain conformations with the native folded structure. MD simulations starting from the native structure resulted in spontaneous partial unfolding and refolding events. The simulation studies indicate that the conformation of the Trp6 residue has a critical influence on the folding process. Although of similar average initial deviation from the native structure only intermediate collapsed conformations with the Trp6 residue in a near-native rotameric state folded rapidly within <100 ns to the native structure. Stable folding of start structures with Trp6 in the non-native rotameric state required an unfolding followed by a transition of Trp6 to the native rotameric state which then rapidly folded to the native structure. Interestingly, fully extended start structures with the Trp6 residue restrained to the native rotameric side chain conformation folded rapidly in <250 ns whereas the same starting structures did not reach a near-native folded state within 1000 ns (1 µs). The results indicate that the conformation and interaction of a single side chain creates a barrier for folding and has a significant influence on the transition to near natively folded states of the Trp-cage protein. Such effects might be of general importance for other protein folding processes.

## Materials and Methods

### Simulation Setup

Three different sets of unrestraint continuous molecular dynamics (cMD) simulations were carried out starting from native, extended and intermediate structures of the trp-cage protein. Native simulations were started from the folded conformation of the protein taken from the pdb-entry 1Y2R (1^st^ structure of the NMR ensemble) [1]. In the case of simulations starting from fully extended conformations, an initial extended Trp-cage structure was generated using the *xleap* module of the Amber package [40]. Intermediate simulations were started from a set of 15 intermediate structures that were obtained from our previous [30] BP-REMD [41] simulation (see below). The *Xleap* module was used to add missing hydrogen atoms and to add a Cl- ion to neutralize the systems. All systems were solvated with TIP3P [42] water molecules to form a truncated octahedral box with at least 10 Å between solute atoms and the borders of the box. The systems were prepared for using the Amber force fields ff03 [43], ff99SB [44], ff99SB_ILDN [45] and ff99SB_NMR [46]. All simulation systems were subjected to energy minimization (5000 steps) using the sander module of Amber11. During MD simulation the protein was initially harmonically restrained to the energy minimized start coordinates and the system was heated up to 300 K in three steps followed by gradual removal of the positional restraints and 0.5 ns unrestrained equilibration at 300 K. All MD simulations were carried out in explicit solvent at 300 K, long range electrostatic interactions were treated with particle mesh Ewald method [47] using a real space cutoff distance of 9 Å. The *Settle* [48] algorithm was used to constrain bond vibrations involving hydrogen atoms, which allowed a time step of 2 fs. Simulation trajectories were visualized using VMD [49] and figures were generated using pymol [50].

### Unrestrained Production Simulations

The resulting systems were used as starting structures for the respective production runs. In the case of “native” simulations, cMD simulations were carried out for 100 ns starting from the experimental folded structure. In the second set five independent simulations (with different initial velocities) under each force field were started from completely extended structures and cMD simulations were carried out for 500 ns. In the third set 15 different simulations were carried out for 100 ns starting from 15 different intermediate structures that were identified from a previous Trp-cage folding simulation study [30]. Details of various simulations are summarized in Table 1.

**Table 1:** Simulation details.

Force field	Simulation time (ns)				
	Unrestraint			Restraint^a^	
	Native start structure	Extended startstructure	Intermediate start structure	Extended startstructure	Intermediate start structure
ff03	1×1001×500^b^	5×5001×1000	15×100	1×250	1×100
ff99SB	1×100	5×5001×1000	15×100	1×250	1×100
ff99SB_ILDN	1×100	5×500	15×100	–	1×100
ff99SB_NMR	1×100	5×500	15×100	–	3×100

a Side chain dihedral angels of the Trp6 residue was weakly restrained towards the conformation in the native folded structure (see Methods).

b Simulation started from native structure but with the side chain dihedrals of Trp6 residue in non-native side chain conformation (flipped by 180° from its native rotameric state).

### Intermediate Starting Structures

All the intermediate structures that are used in this study were identified from our previous study [30] on folding of Trp-cage protein employing a Hamiltonian replica exchange MD approach based a backbone biasing potential along the replica runs (BP-REMD) [41]. The purpose of the biasing potential in these simulations was to reduce the energy barrier associated with the peptide backbone dihedral transitions. With this approach it was possible to successfully fold Trp-cage protein using a small set of replicas. Folding simulations typically resulted in a non-uniform distribution of conformations vs. selected collective variables such as Rmsd or fraction of native contacts. For the Trp-cage protein in a plot of total RMSD with respect to the native structure and fraction of native contacts two highly populated regimes corresponding to folded and unfolded states could be identified. Intermediate structures were randomly picked from the regime between the highly populated parts of the distribution corresponding to RMSD values of 2–6 Å and fraction of native contacts of 40–80%. The conformations were chosen to span the relevant conformational regime (RMSD: 2–6 Å) in a best possible way with a maximum pair-wise RMSD equivalent to partitioning into a set of 15 clusters with pair wise RMSD of ∼2–3 Å. Most of the structures also included a partially of completely formed N-terminal near-native α-helix.

### Simulations Including Dihedral Angle Restraints on the Trp6 Side Chain Conformation

To investigate the influence of the folding process on the conformation of the Trp6 side chain harmonic restraints were included to keep the side chain dihedrals χ1 and χ2 within a window of +/−10° to 180° and 270°, respectively, observed in the native trp-cage structure (force constant for deviations >10° was 2 kcal mol^−1^deg^−2^). Restrained simulations were carried out starting from fully extended structures and from intermediate collapsed structures as indicated in Table 1 and in the Results section.

## Results and Discussion

### Simulations Starting from the Native Trp-cage Structure

Starting from the experimental Trp-cage structure (pdb1Y2R) cMD simulations with four different force fields were carried out for 100 ns at 300 K (Table 1). Overall in all simulations under all force field conditions the sampled structures remained close to the experimental start conformation (Figure 1). However, in several cases occasional large root mean square deviations (RMSD_Cα_) of up to ∼3 Å from the native structure were observed (Figure 1B). These fluctuations corresponded largely to a single type of partial unfolding and refolding of the C-terminal PolyPro II (PP_II_) segment (illustrated in Figure 1D) dissociating from the hydrophobic core (transient disruption of the Trp6-Pro18 contact in the folded state). Conformational variability of the C-terminal segment has also been observed in the published ensemble of NMR structures [1]. Interestingly, a different orientation of the C-terminal segment relative to the N-terminal α-helix has been indicated as main difference between an intermediate and the folded structure in a T-jump/IR study [9]. In addition, in all four different force field simulations the salt bridge between Asp9 and Arg16 showed significant fluctuations and frequent disruption and reformation (Figure 1C). This has also been reported in previous MD simulation studies [14], [21], [30].

### Simulations Starting from Fully Extended States

**Figure 2:**
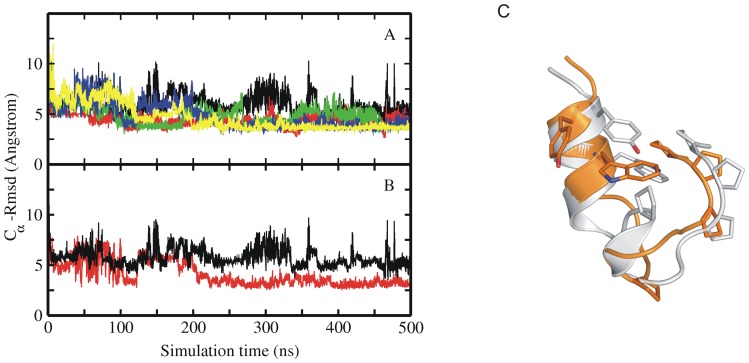
MD simulations of extended Trp-cage structure. (A) RMSD_Cα_ of sampled conformations during five cMD simulations with different atomic velocities starting from an extended Trp-cage structure (A). Simulation results in A were obtained using the ff03 force field. (B) Comparison of the RMSD_Cα_ of sampled structures for one simulation with respect to a partially unfolded conformation (red curve) and with respect to the native Trp-cage (black curve). The partially unfolded conformation was obtained in simulations starting from the native Trp-cage structure and contains a partially dissociated C-terminal segment (see Figure 2C). (C) Example snapshot of a collapsed structure (orange Cartoon) that came close to within 2.5 Å RMSD_Cα_ with respect to the partially unfolded structure (grey Cartoon in C). Residues important for folding (Tyr3, Trp6, Pro12, Pro17, Pro18 and Pro19) are shown as sticks.

Continuous MD simulations were started from a fully extended Trp-cage protein structure generated using the Amber *leap* module. Five independent runs (for each force field resulting in 20 independent simulations, Table 1) using different initial velocity assignments were carried out for 500 ns at 300 K and conformations were stored for every 4 ps. After an initial rapid reduction in RMSD_Cα_ (from ∼12 Å to ∼6 Å, and even down to 3.5 Å) the sampled conformations remained on average in a collapsed state but did not reach near native structures with RMSD_Cα_ <3 Å (Figure 2A). This was found for all force fields (Amber ff03, shown in Figure 2, not shown for ff99SB, ff99SB_NMR and ff99SB_ILDN). The observation of a largely collapsed unfolded state agrees with both experimental and other computational studies [5]–[15], [26], [29]–[31]. Interestingly, however, the sampled structures in these simulations occasionally approached the backbone conformation of the partially unfolded states observed during the MD simulations starting from the native Trp-cage protein (discussed in the previous paragraph) quite closely (Figure 2B,C). In the simulations starting from the native structure these backbone states folded rapidly back into the native conformation (within few ns, Figure 1B) but not if started from the extended structures. Instead, the conformations are trapped in the intermediate state for significant simulation times. Hence, there must be another important conformational difference with respect to the native structure that prevents the transition to the native conformation (with a final RMSD_Cα_ <2 Å). As indicated below one main component of this difference corresponds to the side chain conformation of the Trp6 residue. The Trp6 dihedral side chain conformation adopted only infrequently the native rotameric state during the simulations that resulted in unfolded collapsed states (∼10%, see Figure S1) and remained in one rotameric state for long periods of simulation time (hundreds of ns, Figure S1). Besides Trp6, there is only one other large aromatic side chain, Tyr3, that participates in the hydrophobic core of the Trp-cage protein. In contrast to Trp6, this side chain adopted mainly the native rotameric state (>70%) during the 500 ns simulation time starting from extended Trp-cage conformations (and also undergoes frequent transitions).

**Figure 3:**
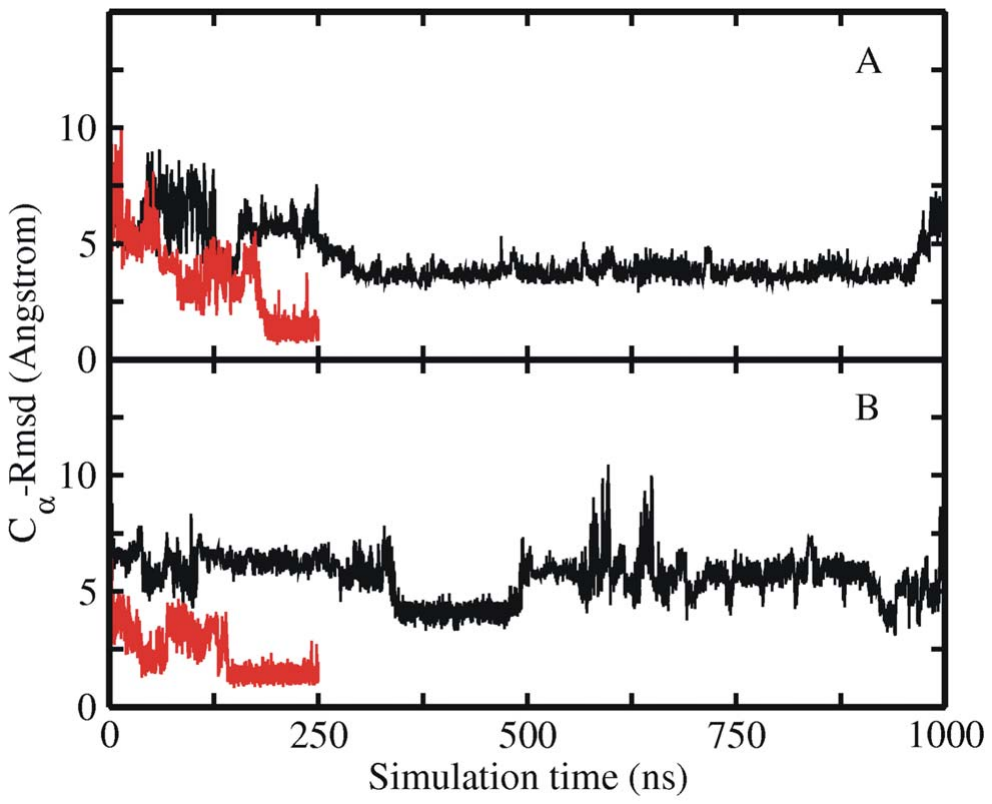
Free and restrained MD simulations of extended Trp-cage structure. RMSD_Cα_ of sampled Trp-cage conformations in an unrestraint explicit solvent MD simulation starting from an extended Trp-cage conformation (black line) and including dihedral angle restraints of the Trp6 residue (red line). (A) Both simulations were started from the same start structure and initial velocities employing the ff03 force field. (B) Same as in (A) but using the ff99SB force field (instead of ff03).

In each of the four different force fields part of the native secondary structure was already observed in most of the cMD simulations although the folded state was not reached. In particular the N-terminal α-helix formed by residues 2–8 in the folded trp-cage protein was partially formed in most of the cMD simulations starting from extended states (Figure S2). This agrees with several experimental results [5]–[15] which provide evidence for the existence of the N-terminal α-helix, already in the collapsed intermediate state. Extension of simulations up to 1 µs did not result in the folded conformation [black lines in Figure 3]. In long cMD simulations of the Trp-cage protein by Lindorff-Larsen et al. [35] close to the melting temperature of the protein and a mean folding time of 12 µs was observed. This time, although obtained with a different force field, is considerably larger than the simulation times in the present study and is qualitatively consistent with the absence of folding events on the present time scales ∼1 µs.

As already discussed above the collapsed but non-native states of Trp-cage with a RMSD_Cα_ of 3–4 Å must differ from the native structure in some important aspect that prevents rapid folding (one may call this a pre-transition state ensemble). In order to identify these key elements we performed several simulations starting from several intermediate structures which indicate that the orientation of the side chain of Trp6 is an important factor for rapid folding. To systematically investigate the importance of conformation/orientation of the Trp6 side chain we carried out simulations with restrains on the side chain dihedrals of the Trp6 residue. Starting again from a fully extended structure two independent simulations (using ff03 and ff99SB force fields, respectively) were carried out for 250 ns with the Trp6 χ1 and χ2 side chain dihedrals restrained to stay close to the native rotameric state in the folded structure (see Methods section for details, Table 1). Interestingly, both simulations reached the folded state of the protein with a RMSD_Cα_ ∼1.5 Å already after ∼200 ns time (red lines in Figure 3A, B). The short folding time suggests a folding with significantly reduced barrier. As indicated already, unrestrained simulations started from the same initial conditions extended to 1000 ns did not reach the folded state (Figure 3A, B).

### Simulations Starting from Various Intermediate Conformations

**Figure 4:**
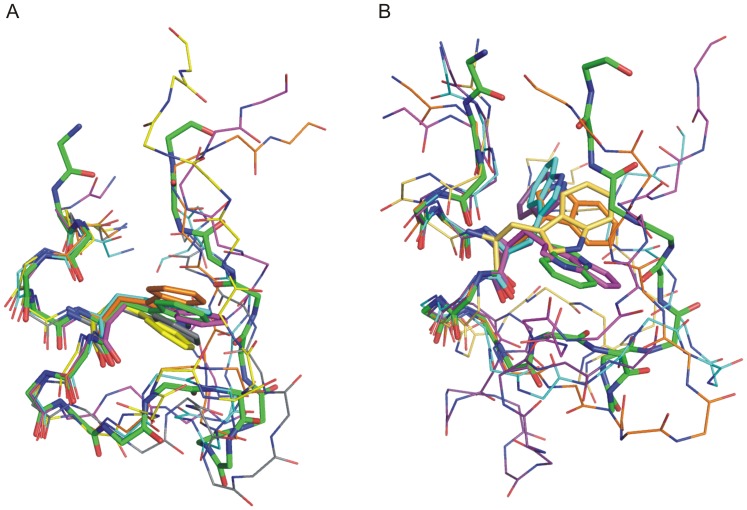
Structural superposition of Set1 and Set2 intermediate start structures. Superposition of (A) set 1 and (B) set2 intermediate start structures (superimposed on α-helix of the native Trp-cage structure indicated as atom colored stick representation). The intermediate start structures are shown as thin sticks using different colors. For clarity only the side chain of the central Trp6 residue in each structure is shown explicitly (stick representation).

MD simulations were also started from 15 “intermediate” Trp-cage structures that were obtained in previous BP-REMD folding simulation studies [30] (Table 1, 2). Each structure was re-equilibrated before starting 100 ns MD simulations employing the four different force fields (see Methods for details). The intermediate structures were all in collapsed states with an Rg and RMSD close to what was observed as average Rg and RMSD of conformations obtained in the simulations starting from fully extended structures. Each starting structure differed, however, in the degree of initial near native structure and topology. In one set of intermediate structures (termed set 1) in addition to the N-terminal α-helix, other important native secondary structural elements such as the 3_10_ helix and the C- terminal PP_II_ helix were partially formed (Figure 4A). Some of these intermediate structures are overall similar to the topology seen for partially unfolded structures observed during simulations of the native Trp-cage structure (Figure 1) with a RMSD_Cα_ <3 Å from the native conformation and importantly with the Trp6 residue in the near native rotameric state (Table 2). For the start structures and all force fields rapid folding was observed within 100 ns down to RMSD_Cα_<∼1.5 Å (Figure S3, Table 2) and most of the important structural features seen in the experimental Trp-cage structure were formed. This includes also the salt bridge between Asp9 and Arg16 which was formed but with frequent fluctuations. One can conclude that the set 1 starting conformations are within an “attractive basin” of the folded state and there is no significant barrier left with respect to the folded conformation (post-transition state ensemble).

**Table 2:** Results of simulations starting from different intermediate state of Trp-cage.

S. No	Starting RMSD_Cα_ (Å)	Final RMSD_Cα_ (Å)				Final state
		ff03	ff99SB	ff99SB_ILDN	ff99SB_NMR	
**Set1**						
1	2.8	0.9	1.0	0.8	0.9	Folded
2	3.1	0.8	1.0	0.9	0.8	Folded
3	1.8	0.8	0.9	1.0	0.85	Folded
4	3.2	1.0	1.1	1.2	1.1	Folded
5	2.9	0.9	0.9	1.0	0.9	Folded
**Set2**						
6	3.9	0.8	0.8	1.0	1.1	Folded
7	4.0	1.0	1.2	1.2	1.1	Folded
8	4.9	0.9	1.2	1.1	1.0	Folded
9	5.5	1.1	1.3	1.2	0.9	Folded
10	4.5	1.0	0.9	1.0	1.0	Folded
11	3.8	2.9	3.9	3.4	3.5	Misfolded
12	4.1	4.5	4.4	4.3	4.5	Collapsed
13	5.0	6.2(1.0)	6.0(0.8)	6.5(1.9)	6(0.9)	Unfolded (folded)
14	4.0	7.0	6.5	5.5	6(1.2)	Unfolded (folded)
15	3.5	4.0	5.0	4.5	3.5(1.0)	Collapsed (folded)

Values in brackets correspond to the results of simulation with restraints on the side chain dihedral angles of the Trp6 residue to keep it close to the conformation in the native folded structure (see Methods for details). Start conditions for unrestraint and restraint simulations were the same. The Trp6 residue was in its native rotameric state in all the Set1 intermediate start structures and in non-native rotameric state in all the Set2 intermediate start structures.

**Figure 5:**
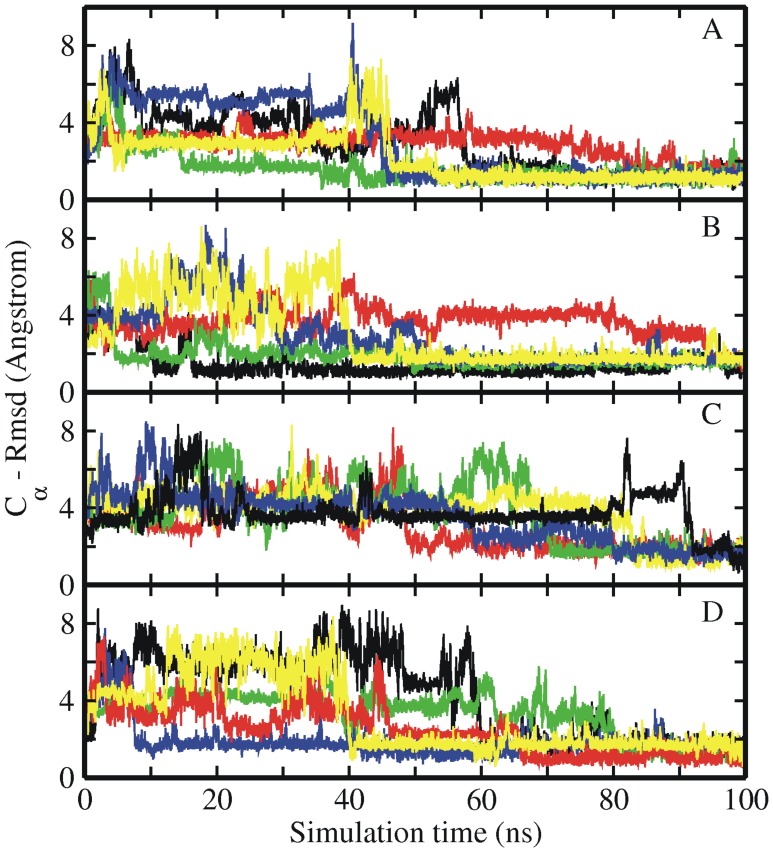
MD simulations of intermediate Trp-cage structures. RMSD_Cα_ of sampled Trp-cage conformations in explicit solvent starting from a set of intermediate structures (a subset of intermediate structures of set2 shown in Figure 5B,) vs. simulation time with different Amber force fields (A) ff03 (B) ff99SB, (C) ff99SB_ILDN, (D) ff99SB_NMR. All these examples eventually reached a near-native conformation with RMSD_Cα_ <2 Å from the native Trp-cage structure.

**Figure 6:**
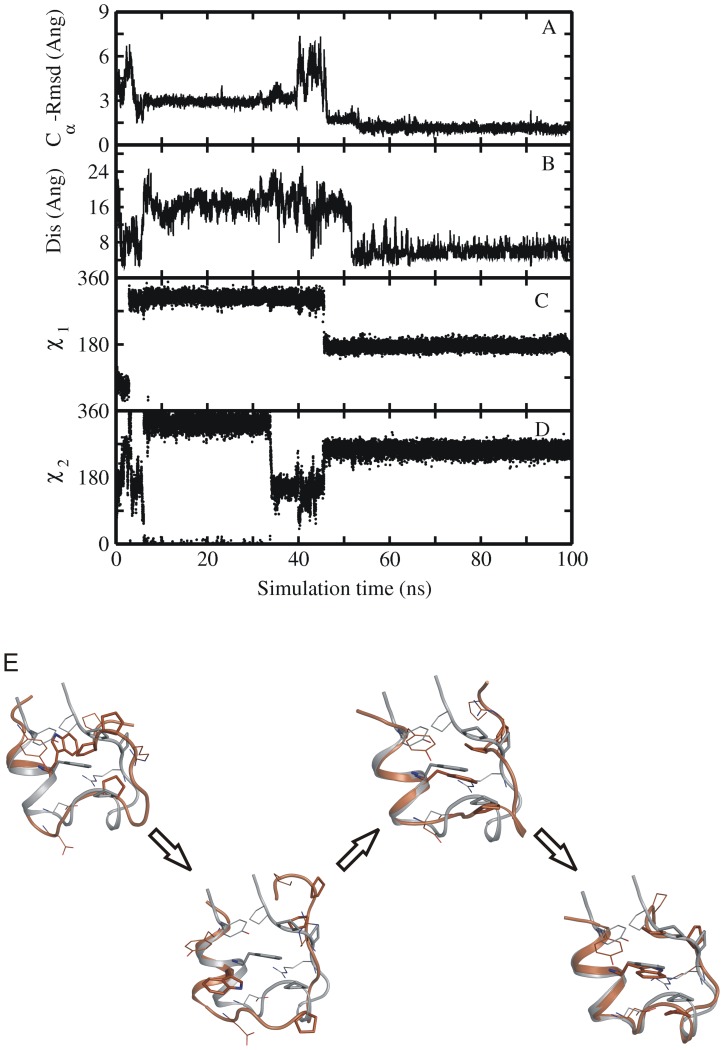
Time-dependence of backbone Rmsd and Trp6 side chain dihedral angles. (A) RMSD_Cα_ from native structure and (B) Asp9-Arg16 salt bridge distance as well as (C, D) side chain dihedral angels (χ1 and χ2) of Trp-6 residue of sampled Trp-cage conformations along one folding trajectory starting from a set2 intermediate structure vs. simulation time (force field ff03). The dihedral angles of the native Trp6 side chain correspond to χ1 in trans (∼180°) and χ2 in –gauche (∼270°) (E) MD simulation snapshots showing the various steps in the folding process of the protein, as in the order of occurrence. Simulation snapshots (gold ribbons) are superimposed onto the native structure (grey ribbons), and key residues (Tyr3, Trp6, Ala9, Pro12, Arg16 and Pro17-19) are shown as sticks (same color coding as for ribbons).

**Figure 7:**
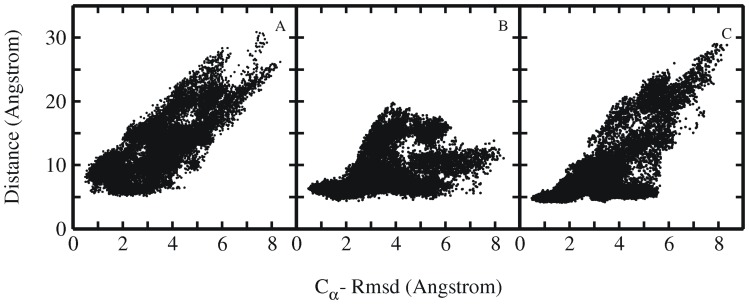
Rmsd and residue pair distance distribution. Distribution plots showing the correlation between RMSD_Cα_ and distance between the residue pairs (A) Tyr3-Pro19, (B) Trp6-Pro12 and (C) Trp6-Pro18. Each point represents one sampled state in all simulations starting from intermediate Trp-cage structures.

In another set of intermediate structures (set 2) only the N-terminal α-helix was already partially formed, but the rest of the protein differed significantly from the native structure missing the 3_10_ helix, part of the PP_II_ helix and proper native tertiary contacts (Figure 4B, Table 2). These intermediate structures had an RMSD_Cα_ of 3.5–6.0 Å from the experimental structure and in most cases Trp6 was in a non-native rotameric state (Figure 4B, Table 2). In contrast to the first set only a part of the start structures from the second set of intermediate conformations reached the folded state of the Trp-cage protein within 100 ns MD simulation time (Figure 5, Table 2). In some of the simulations the Trp-cage conformation remained close to the start structure and also unfolding of the protein was observed (Figure S4, Table 2). Detailed analysis of simulation trajectories that reached the folded state of the protein revealed some interesting key events that occurred along the folding pathway. Interestingly, in most of the simulations that resulted in folding to near-native conformations the protein eventually started to move away from its compact start state (Figure 5, 6). The final transition to the folded conformation was typically associated with a sudden drop of the RMSD_Cα_ from ∼3–7 Å down to ∼<1.5 Å. A prerequisite for successful collapse to the native structure was either the presence of a near-native rotameric state of the Trp6 side chain or the transition of the Trp6 side chain to the native rotameric state typically several nanoseconds before the successful collapse to the near-native conformation (indicated for one case per force field in Figure 6 and in Figures S5, S6, S7). In the majority of successful folding events, the Trp6 first establishes interaction with the Pro12 residue (in the middle 3_10_ helix segment). Subsequently, contacts of Trp6 with Pro18 or Pro17 from the PP_II_ motif at the C-terminus are established to complete the process (Figure 6E, and Figure 7). Successful folding to low final RMSD_Cα_ is especially tightly coupled to contacts between Trp6 and Pro12 as well as Pro18 (there are no near-native Trp-cage structures without close Trp6-Pro12 and Trp6-Pro18 contacts, Figure 7). There is no such tight coupling between the Tyr3-Pro19 distance and occurrence of near –native Trp-cage structures. Even for sampled conformations with RMSD_Cα_ <2 Å from the native structure the Tyr3-Pro19 distance can vary between 5–12 Å (Figure 7A).

**Figure 8:**
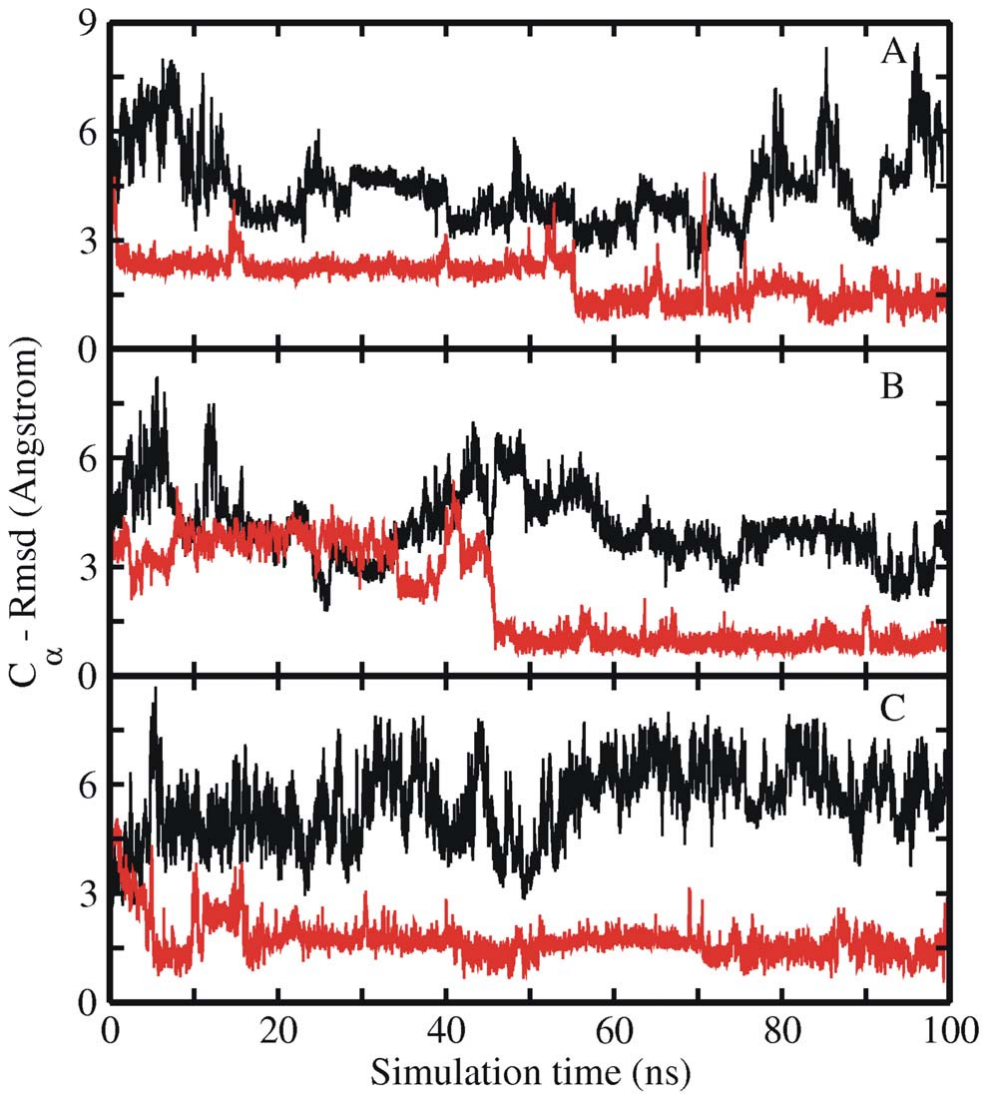
Free and restrained MD simulations of intermediate Trp-cage structure. RMSD_Cα_ with respect to the native Trp-cage structure starting from an intermediate state (of set 2) with (red lines) and without (black lines) side chain dihedral angle restraints on Trp6 residue vs. simulation time. Results are shown for three different force fields (A) ff03, (B) ff99SB and (C) ff99SB_NMR.

In order to further check the importance of the Trp6 rotameric state for the speed of the final Trp-cage folding, simulations in the presence of side chain dihedral restraints of the Trp6 to keep it close to the native rotameric state were performed starting from a set 2 start structure that didn’t reach the folded state within 100 ns time (similar to the simulations starting from extended structures, see above). As a result of dihedral angle restraints, the set 2 starting structures folded successfully to near-native structures within 100 ns, while the same structures didn’t reach the correctly folded state during 100 ns unrestrained simulation (compare red and black RMSD_Cα_ curves in Figure 8 and summarized results in Table 2).

**Figure 9:**
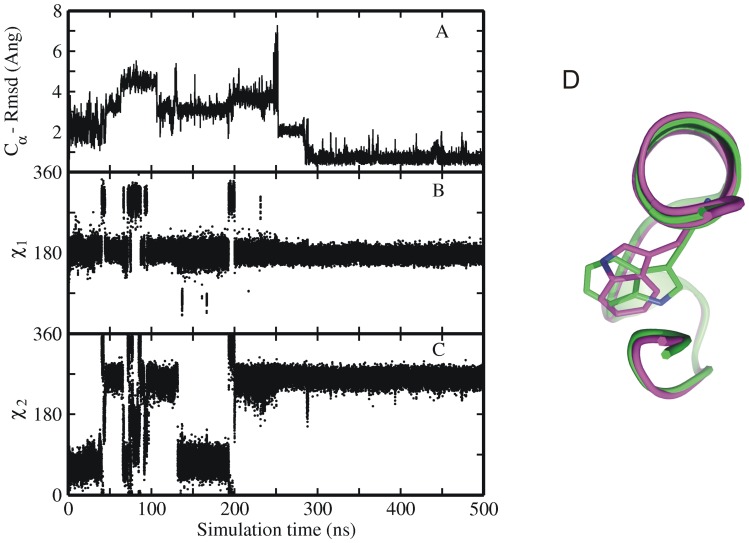
MD simulation of native Trp-cage structure with non-native Trp6 orientation. (A) Root-mean –square deviation (RMSD_Cα_) and (B, C) side chain dihedral angels of Trp6 residue of sampled Trp-cage conformations in explicit solvent starting from native structure with Trp6 residue in incorrect orientation versus simulation time with force field ff03. (D)The right panel shows the superposition of two examples of Trp-cage structures with the Trp6 side chain in the native conformation (green, χ2∶270°) and the in a flipped (incorrect, χ2∶60°) conformation (magenta). Average values of side chain dihedrals angels (χ1 and χ2) of Trp-6 residue of sampled Trp-cage conformations correspond to 180° and 270°.

One interesting non-native compact structure was sampled with Trp6 partially buried in the hydrophobic core but with the χ2 dihedral angle in the incorrect (∼60° instead of ∼270°) orientation (Figure 9). To investigate the stability of this non-native compact structure with incorrect conformation of the Trp6 residue, simulations were carried out starting from the native structure but with the incorrect side chain dihedrals of Trp6 residue (Figure 9D). The RMSD_Cα_ of this structure reached 2.5–3 Å with respect to the native Trp-cage conformation, already at very early stage of simulation and the simulation of this structure was extended to 500 ns. However, the conformation was only transiently stable and eventually underwent a transition to the natively folded conformation (Figure 9). Similar to other collapsed intermediate start structures this transition required a partial unfolding to allow the side chain to flip into the native conformation (increased RMSD_Cα_ from the native structure of ∼4–5 Å in the time range of 50–200 ns in Figure 9A) followed by a collapse to a stable near-native structure at 300 ns (with a RMSD_Cα_ <2 Å). This collapse was preceded by a flip to the correct Trp6 rotameric state (Figure 9B, C) approximately ∼70 ns before the final folding.

### Analysis of Salt-bridge Formation

**Figure 10:**
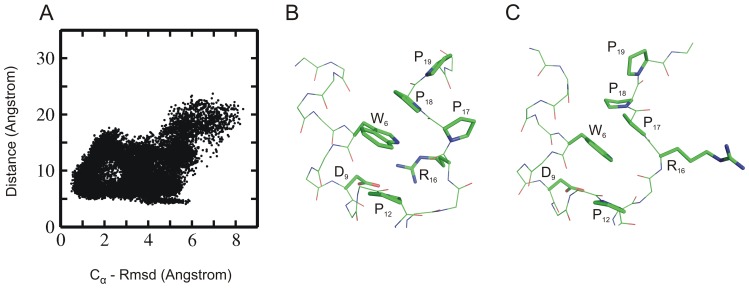
Rmsd and salt bridge distribution. (A) Distribution plot showing the correlation between RMSD_Cα_ (from native structure) and the salt-bridge distance between residues Asp9– Arg16 of sampled Trp-cage conformations (starting from intermediate states simulated using Amber 03 force field). (B) Example snapshot with a small near native salt bridge distance (side chains of residues near Trp6 are shown as sticks with labels, backbone as thin sticks). In this conformation the Pro18 stacks on the side chain of Trp6. (C) Example of a near-native sampled state with large Asp9-Arg16 distance and non-native stacking of Pro17 (instead of Pro18) onto the Trp6 side chain.

The salt bridge between Asp9– Arg16 observed in the native folded Trp-cage structure was also formed transiently in several simulations starting from extended or collapsed intermediate states. However, overall no significant correlation between salt bridge formation and backbone deviation of the sampled states from the native fold was observed (Figure 10A). As the RMSD_Cα_ of the sampled conformations reached ∼1.5 Å, the salt bridge distance between the Asp9 and Arg16 was still larger than 10.0 Å. However, transient formation of salt bridge was seen once the simulated system reached the near native state (with RMSD_Cα_ ∼1.5–2 Å). This agrees with the rapid fluctuations of the salt-bridge seen in the simulations that started from the native structure (Figure 1C). Contradictory to several previous simulation studies [17], [18], [20], [23], [26], [34] that often employed implicit solvent models, our results indicate that the formation of the salt bridge is not a prerequisite for the proper folding of the mini protein. And in agreement with experimental findings [8], [11], [12], [14], the present explicit solvent folding simulations indicate that salt bridge could form at a later stage of the folding process. However, in the absence of salt bridge an alternatively folded conformation was sampled as second most populated state (∼10% folded state) during the simulations. The accumulation of this state is stabilized by Pro17 stacking on Trp6 instead of Pro18 stacking on Trp6 in the native Trp-cage structure (Figure 10B, C). A similar structure was also observed previously in folding simulations of Trp-cage protein using BP-REMD [22], [30]. It was also sampled in the simulations starting from the native Trp-cage structure (not shown).

## Conclusions

Trp-cage being one of the smallest and fastest folding proteins is also one of the most extensively studied proteins both experimentally and computationally. Although much has been debated about the folding pathway of the Trp-cage mini protein, it is widely accepted now with increasing experimental evidence, that the folding of Trp-cage is not a simple two stage folding mechanism but rather involves semi-stable intermediates along the folding pathway [5]–[9], [14], [15]. Early formation of near-native tertiary contacts (long range hydrophobic) is a major driving force for the collapse of this extended mini-protein to a compact intermediate state, as it is often believed to be a major reason for the fast folding of proteins. Although the initial hydrophobic collapse event was already seen in various successful folding and unfolding studies of the Trp-cage mini protein, the key events that result in final folding are not completely understood. Experimental mutagenesis studies indicate the importance of the central Trp6 residue to the folding and stability of the protein [51]. Substitution by phenylalanine or histidine results in strong destabilization (W6F) or complete unfolding (W6H) of the protein [51]. Previous simulation studies on the Trp-cage protein have employed a range of different force field models to study the folding process. This included coarse-grained or hybrid-resolution representations [38] and atomistic protein representation combined with either implicit solvent models [16]–[22] or explicit solvent models [23]–[35]. It is likely that these force field models differ in the results due to differences in the protein-solvent and side chain-backbone interactions. All these studies have largely focused on the different pathways to the folded topology and have resulted in characterization of backbone intermediate states, the critical role of secondary structure formation in different pathways of folding and the occurrence of native contacts along the folding process. Also, the important role of the Trp6 residue has already been recognized in simulation studies [19], [20] and has been included in the clustering of intermediate states [36], [37]. Experimentally, it is also not possible to capture the movement of key individual residues with high time resolution. To shed more light on the folding mechanism of the Trp-cage protein and to characterize the key role of the central Trp6 residue we employed all atom molecular dynamics simulation. The comparison of four different force fields indicated in all cases formation of intermediate collapsed and semi-compact states after simulations times of less than 500 ns starting from extended conformations. Furthermore, comparative simulations starting from fully extended states indicated a significant acceleration of the folding process if the central Trp6 residue was restraint to its native rotameric state. In addition, it was possible to define a set of intermediate starting structures with most secondary structures at least partially formed and the Trp6 residue in the near-native rotameric conformation that all folded rapidly (in all force fields) within <100 ns. In contrast, only few simulations reached the native state during 100 ns in case of a second set of collapsed structures with Trp6 in a non-native rotameric state. Rapid folding could, however, be induced even for this set if the Trp6 was restraint to its near-native rotamer during the MD simulations. The simulation studies provide qualitative evidence that the rotameric state of a single side chain creates a significant barrier for folding and can act as a control element for the folding process. Many of the rapid collapse events are unsuccessful due to an incorrect Trp6 side chain rotameric state. It determines at least in part the transition from intermediate collapsed states towards the native folded protein structure. It is likely that similar side chain effects play also a key role for the folding of other protein molecules. Previous simulation studies have given partially contradicting results on the role of the Asp9-Arg16 salt bridge on Trp-cage folding. Especially, simulations in implicit solvent emphasized the role of the salt-bridge for the formation and stability of the folded structure [17], [18], [20]. In agreement with experimental findings the Asp9-Arg16 salt bridge was neither well preserved during the simulations (in all force fields), nor is it formed in all the folded conformations that resembles the near-native structure. However, the absence of the salt bridge appeared to be partially coupled to one local backbone conformational change which corresponded to a shift of the C-terminus such that Pro17 instead of Pro18 stacked on the Trp6.

## Supporting Information

Figure S1 **Conformational** **sampling of Tyr3 and Trp6 side chain dihedral angle.** (A,B) Side chain dihedral angels (χ1 and χ2) of Trp-6 residue of sampled Trp-cage conformations along one simulation trajectory starting from an extended structure vs. simulation time (A and B correspond to force field ff03 and ff99SB_NMR, respectively). The dihedral angles of the native Trp6 side chain correspond to χ1 in trans (∼180°) and χ2 in –gauche (∼270°) are highlighted in boxes (black and blue). (C, D) side chain dihedral angels (χ1 and χ2) of Tyr-3 residue of sampled Trp-cage conformations along one simulation trajectory starting from an extended structure vs. simulation time (C and D correspond to force field ff03 and ff99SB_NMR, respectively). The dihedral angles χ1 and χ2 of the native Tyr3 side chain correspond to ∼200° and ∼270° respectively, are highlighted in boxes (black and blue). (E, F) Distribution of side chain dihedrals angles of Trp-6 residue of sampled Trp-cage conformations along one simulation trajectory (same as A and B) starting from an extended state. (G, H) Distribution of side chain dihedrals angles of Tyr-3 residue of sampled Trp-cage conformations along one simulation trajectory (same as C and D) starting from an extended state. In the dihedral distribution plots (E, F, G, H) the native rotameric state of side chain of Trp-6 and Tyr-3 residues are highlighted as red stars. Note, that for Tyr3 side chain conformations with χ2 in the range of 0.180° is sterically equivalent to χ2 in the range of 180.360°. The analysis of side chain dihedral distributions for the two other force fields was similar (data not shown).
(TIF)

Figure S2 **Time evolution of secondary structure.** Evolution of secondary structure of Trp-cage starting from fully extended state during 500 ns continuous MD (cMD) simulations under 4 different force field (ff03:top,ff99SB:second from top,ff99SB_ILDN: second from bottom,ff99SB_NMR:bottom). Secondary structure (blue: α - helix, gray: 3_10_- helix, yellow: turn, green: bend, white: coil) along the protein chain (y-axis) versus simulation time (x-axis).
(TIF)

Figure S3 **MD simulations of Set1 intermediate Trp-cage structures.** RMSD_Cα_ of sampled Trp-cage conformations in explicit solvent starting from a set of intermediate structures (set1 intermediate structures shown in Figure 4A,) vs. simulation time with different Amber force fields (A) ff03 (B), ff99SB, (C) ff99SB_ILDN, (D) ff99SB_NMR.
(TIF)

Figure S4 **MD simulations of set2 intermediate Trp-cage structures.** RMSD_Cα_ of sampled Trp-cage conformations in explicit solvent starting from a set of intermediate structures (a subset of intermediate structures of set2 shown in Figure 4B) vs. simulation time with different Amber force fields (A) ff03 (B), ff99SB, (C) ff99SB_ILDN, (D) ff99SB_NMR.
(TIF)

Figure S5 **Time-dependence** **of backbone** **Rmsd and Trp6 side chain dihedral angles (ff99SB).** (A) RMSD_Cα_ from native structure and (B) Asp9–Arg16 salt bridge distance as well as (C, D) side chain dihedral angels (χ1 and χ2) of Trp-6 residue of sampled Trp-cage conformations along one folding trajectory starting from a set2 intermediate structure vs. simulation time (force field ff99SB). The dihedral angles of the native Trp6 side chain correspond to χ1 in trans (∼180°) and χ2 in –gauche (∼270°).
(TIF)

Figure S6 **Time-dependence** **of backbone** **Rmsd and Trp6 side chain dihedral angles ff99SB_ILDN).** (A) RMSD_Cα_ from native structure and (B) Asp9–Arg16 salt bridge distance as well as (C, D) side chain dihedral angels (χ1 and χ2) of Trp-6 residue of sampled Trp-cage conformations along one folding trajectory starting from a set2 intermediate structure vs. simulation time (force field ff99SB_ILDN). The dihedral angles of the native Trp6 side chain correspond to χ1 in trans (∼180°) and χ2 in –gauche (∼270°).
(TIF)

Figure S7 **Time-dependence** **of backbone** **Rmsd and Trp6 side chain dihedral angles (ff99SB_NMR).** (A) RMSD_Cα_ from native structure and (B) Asp9–Arg16 salt bridge distance as well as (C, D) side chain dihedral angels (χ1 and χ2) of Trp-6 residue of sampled Trp-cage conformations along one folding trajectory starting from a set2 intermediate structure vs. simulation time (force field ff99SB_NMR). The dihedral angles of the native Trp6 side chain correspond to χ1 in trans (∼180°) and χ2 in –gauche (∼270°).
(TIF)
