# Supplementary figures and images for: Role of Tryptophan Side Chain Dynamics on the Trp-Cage Mini-Protein Folding Studied by Molecular Dynamics Simulations
Source: PLoS One. 2014 Feb 7;9(2):e88383. doi: 10.1371/journal.pone.0088383 (PMC3921895; doi:10.1371/journal.pone.0088383)

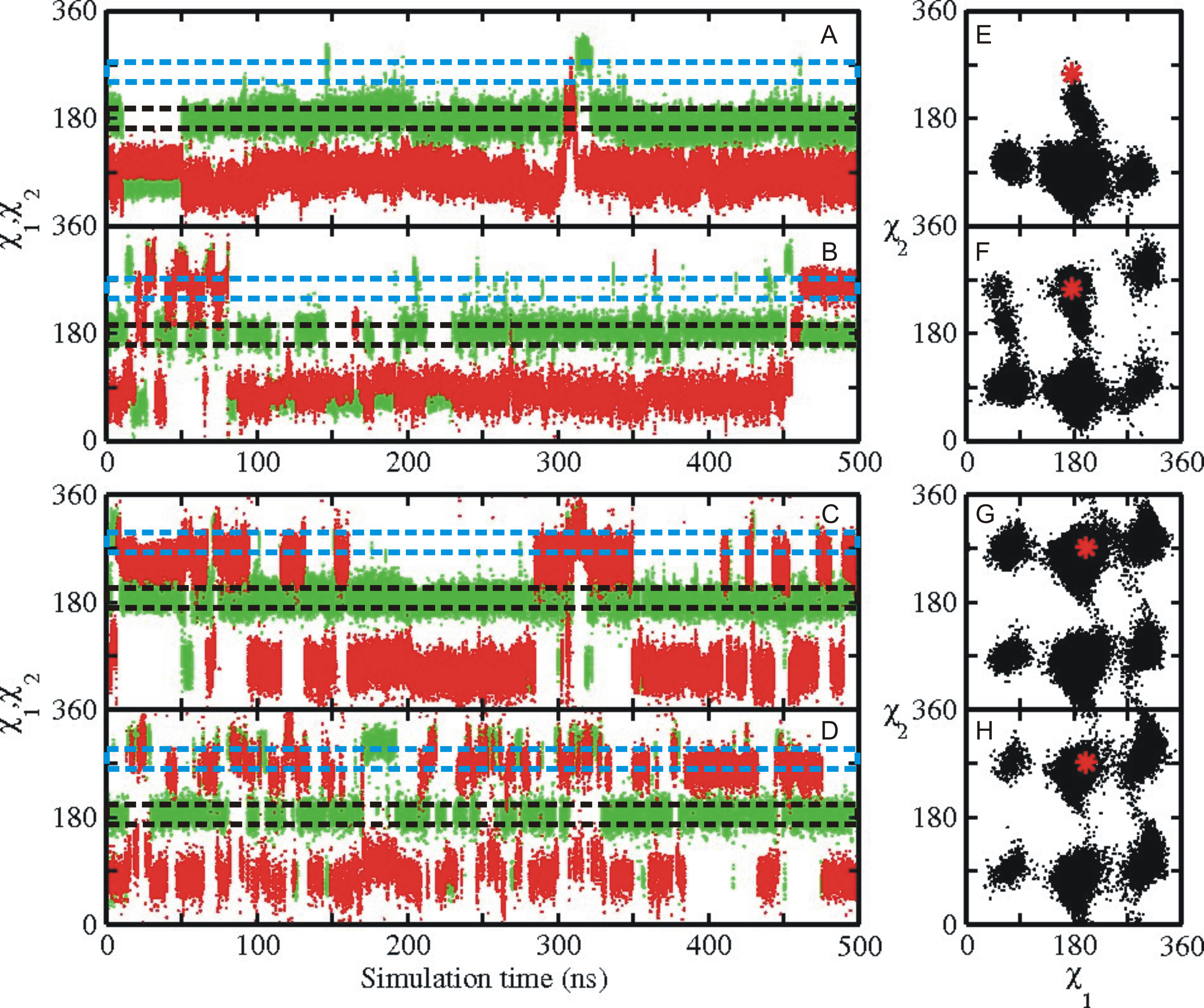

Supplement: Figure S1 — Conformational sampling of Tyr3 and Trp6 side chain dihedral angle. (A,B) Side chain dihedral angels (χ1 and χ2) of Trp-6 residue of sampled Trp-cage conformations along one simulation trajectory starting from an extended structure vs. simulation time (A and B correspond to force field ff03 and ff99SB_NMR, respectively). The dihedral angles of the native Trp6 side chain correspond to χ1 in trans (∼180°) and χ2 in –gauche (∼270°) are highlighted in boxes (black and blue). (C, D) side chain dihedral angels (χ1 and χ2) of Tyr-3 residue of sampled Trp-cage conformations along one simulation trajectory starting from an extended structure vs. simulation time (C and D correspond to force field ff03 and ff99SB_NMR, respectively). The dihedral angles χ1 and χ2 of the native Tyr3 side chain correspond to ∼200° and ∼270° respectively, are highlighted in boxes (black and blue). (E, F) Distribution of side chain dihedrals angles of Trp-6 residue of sampled Trp-cage conformations along one simulation trajectory (same as A and B) starting from an extended state. (G, H) Distribution of side chain dihedrals angles of Tyr-3 residue of sampled Trp-cage conformations along one simulation trajectory (same as C and D) starting from an extended state. In the dihedral distribution plots (E, F, G, H) the native rotameric state of side chain of Trp-6 and Tyr-3 residues are highlighted as red stars. Note, that for Tyr3 side chain conformations with χ2 in the range of 0.180° is sterically equivalent to χ2 in the range of 180.360°. The analysis of side chain dihedral distributions for the two other force fields was similar (data not shown). (TIF) [file pone.0088383.s001.tif]

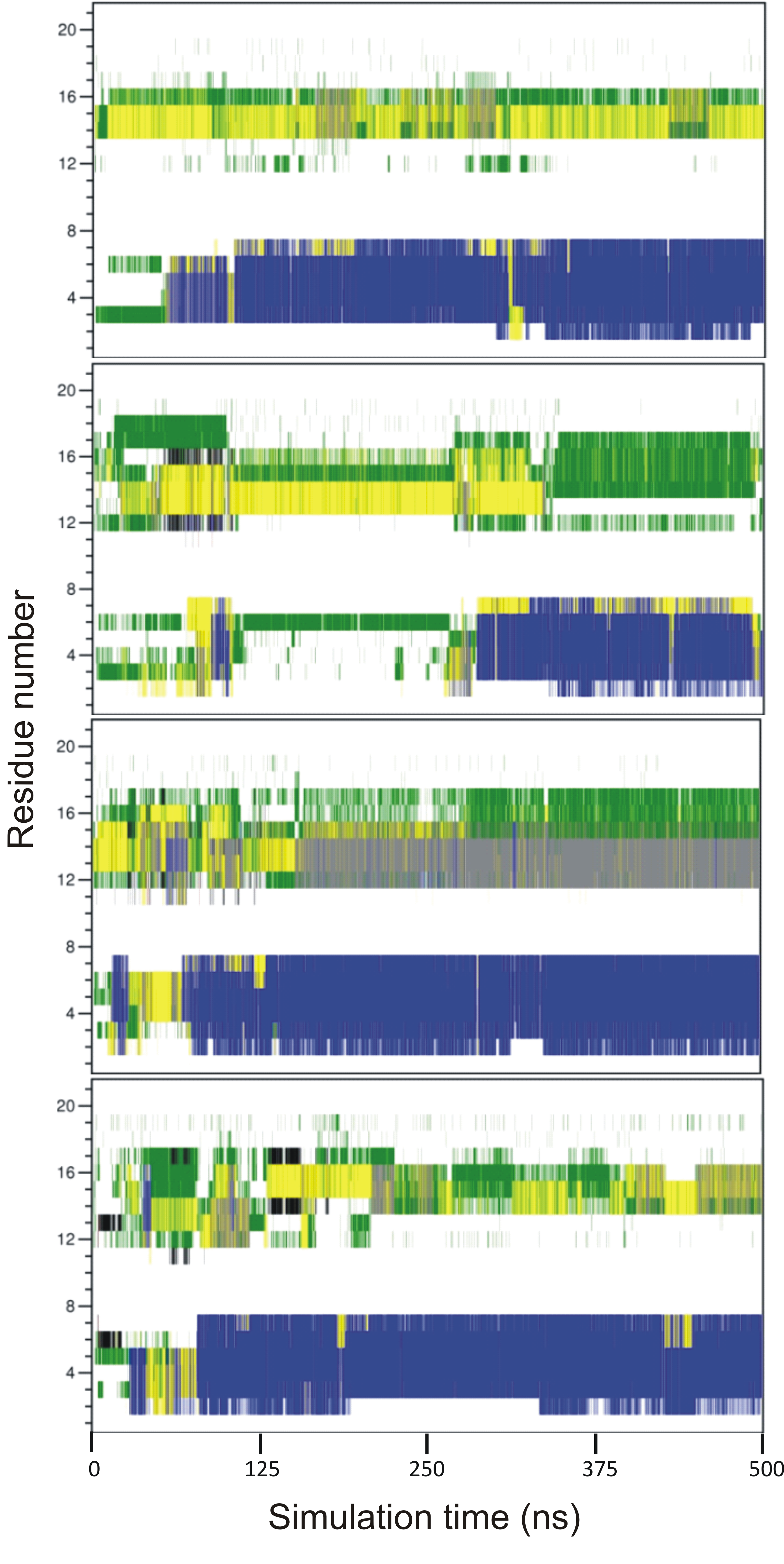

Supplement: Figure S2 — Time evolution of secondary structure. Evolution of secondary structure of Trp-cage starting from fully extended state during 500 ns continuous MD (cMD) simulations under 4 different force field (ff03:top,ff99SB:second from top,ff99SB_ILDN: second from bottom,ff99SB_NMR:bottom). Secondary structure (blue: α - helix, gray: 310- helix, yellow: turn, green: bend, white: coil) along the protein chain (y-axis) versus simulation time (x-axis). (TIF) [file pone.0088383.s002.tif]

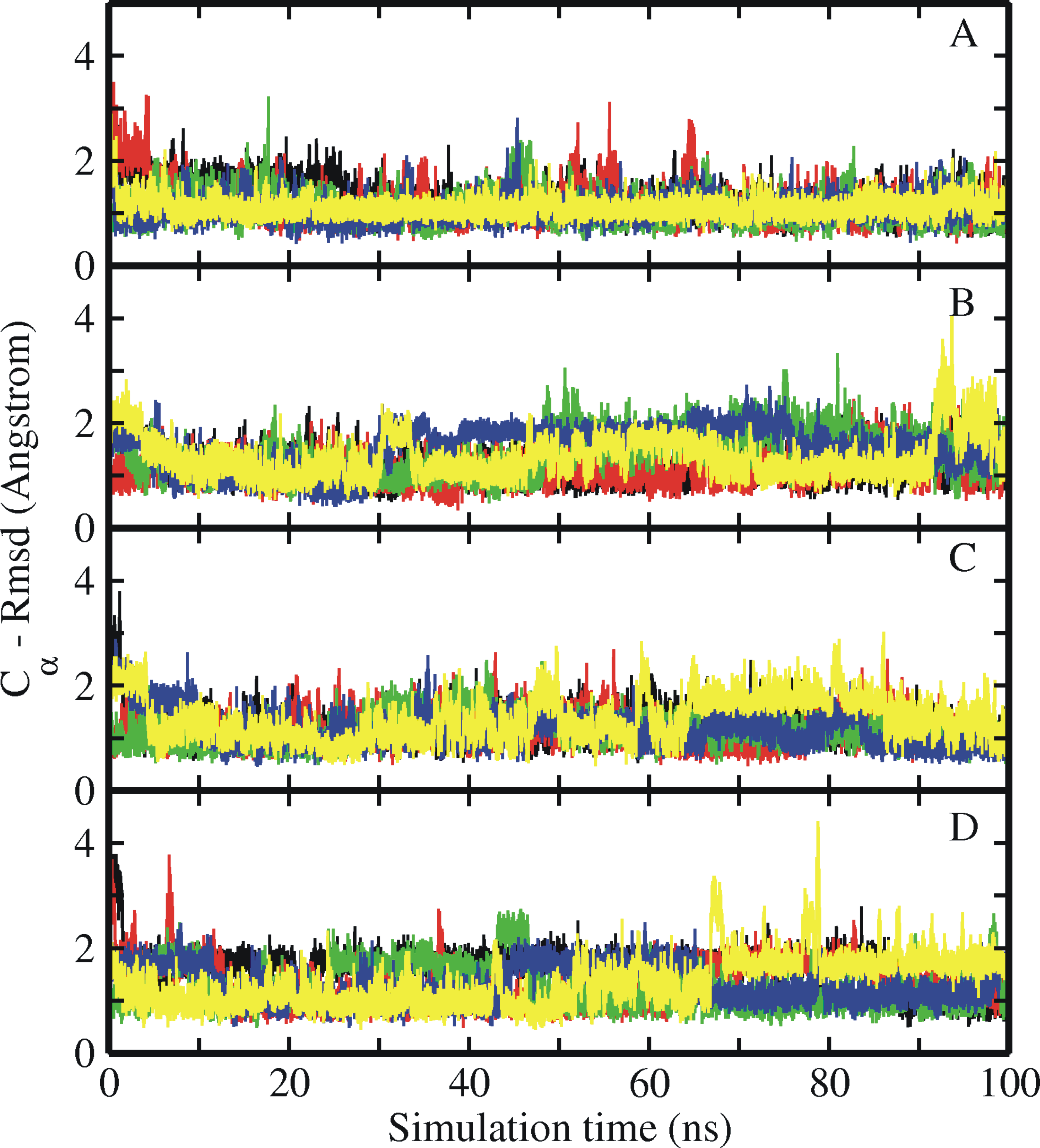

Supplement: Figure S3 — MD simulations of Set1 intermediate Trp-cage structures. RMSDCα of sampled Trp-cage conformations in explicit solvent starting from a set of intermediate structures (set1 intermediate structures shown in Figure 4A,) vs. simulation time with different Amber force fields (A) ff03 (B), ff99SB, (C) ff99SB_ILDN, (D) ff99SB_NMR. (TIF) [file pone.0088383.s003.tif]

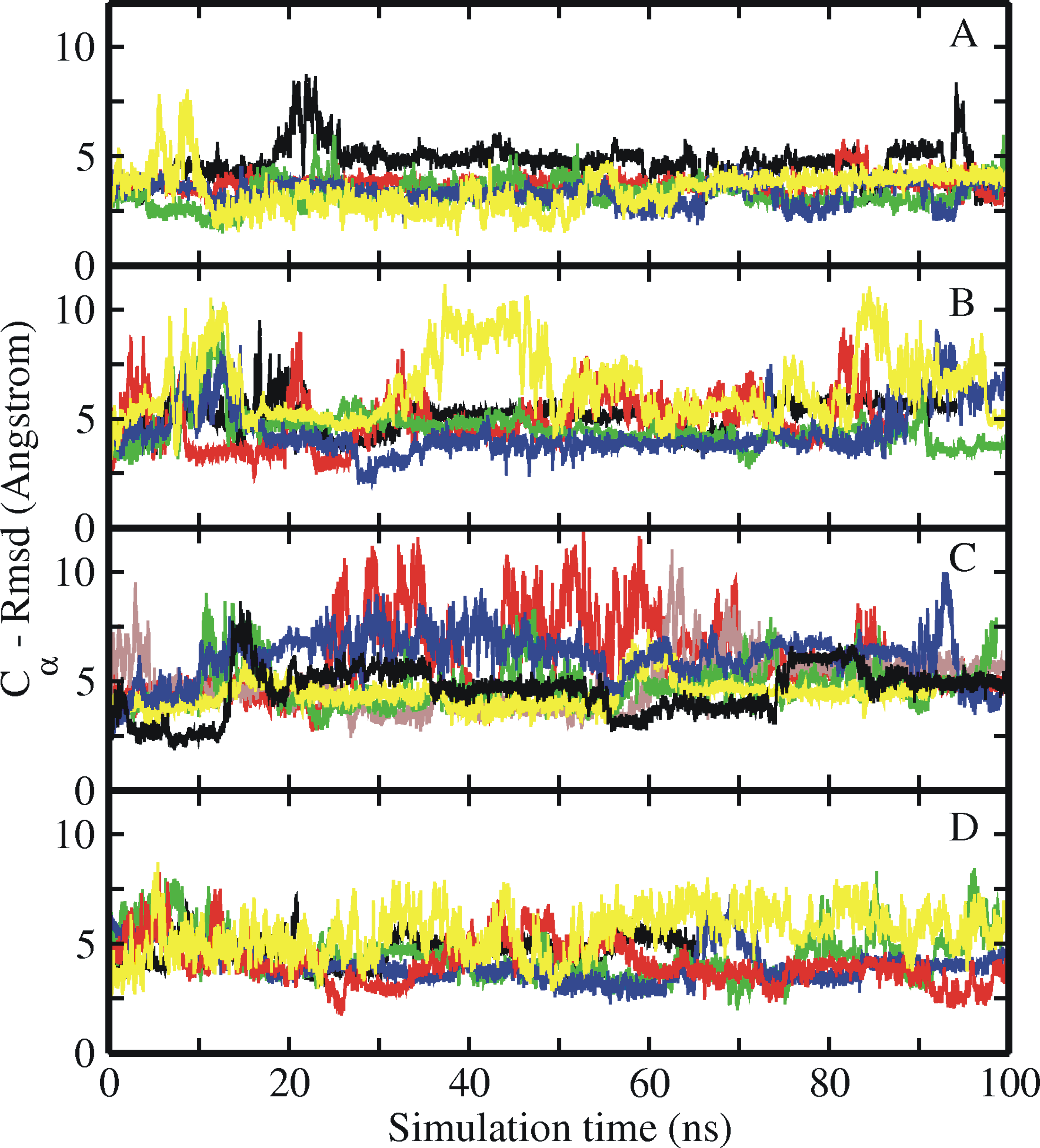

Supplement: Figure S4 — MD simulations of set2 intermediate Trp-cage structures. RMSDCα of sampled Trp-cage conformations in explicit solvent starting from a set of intermediate structures (a subset of intermediate structures of set2 shown in Figure 4B) vs. simulation time with different Amber force fields (A) ff03 (B), ff99SB, (C) ff99SB_ILDN, (D) ff99SB_NMR. (TIF) [file pone.0088383.s004.tif]

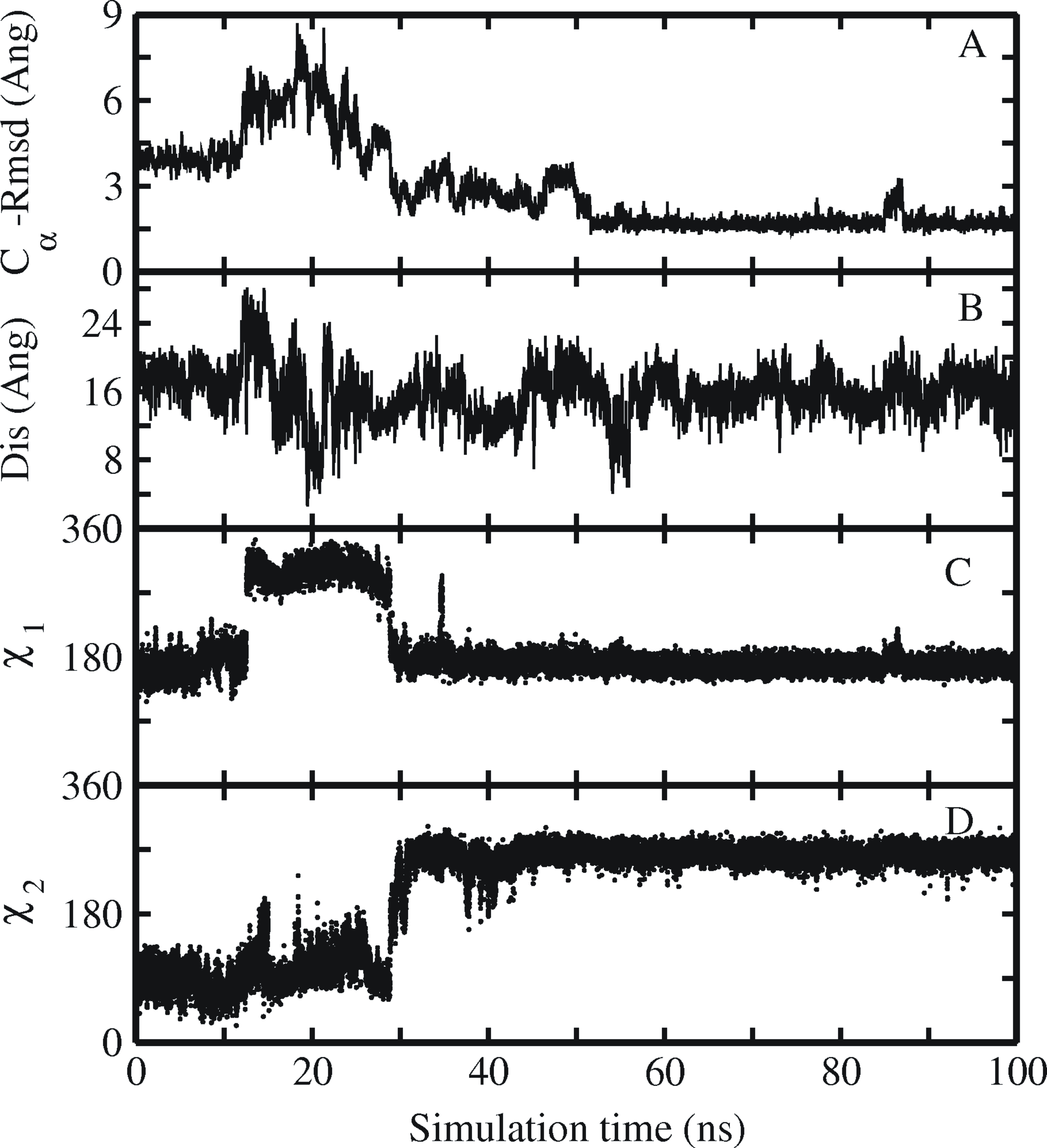

Supplement: Figure S5 — Time-dependence of backbone Rmsd and Trp6 side chain dihedral angles (ff99SB). (A) RMSDCα from native structure and (B) Asp9–Arg16 salt bridge distance as well as (C, D) side chain dihedral angels (χ1 and χ2) of Trp-6 residue of sampled Trp-cage conformations along one folding trajectory starting from a set2 intermediate structure vs. simulation time (force field ff99SB). The dihedral angles of the native Trp6 side chain correspond to χ1 in trans (∼180°) and χ2 in –gauche (∼270°). (TIF) [file pone.0088383.s005.tif]

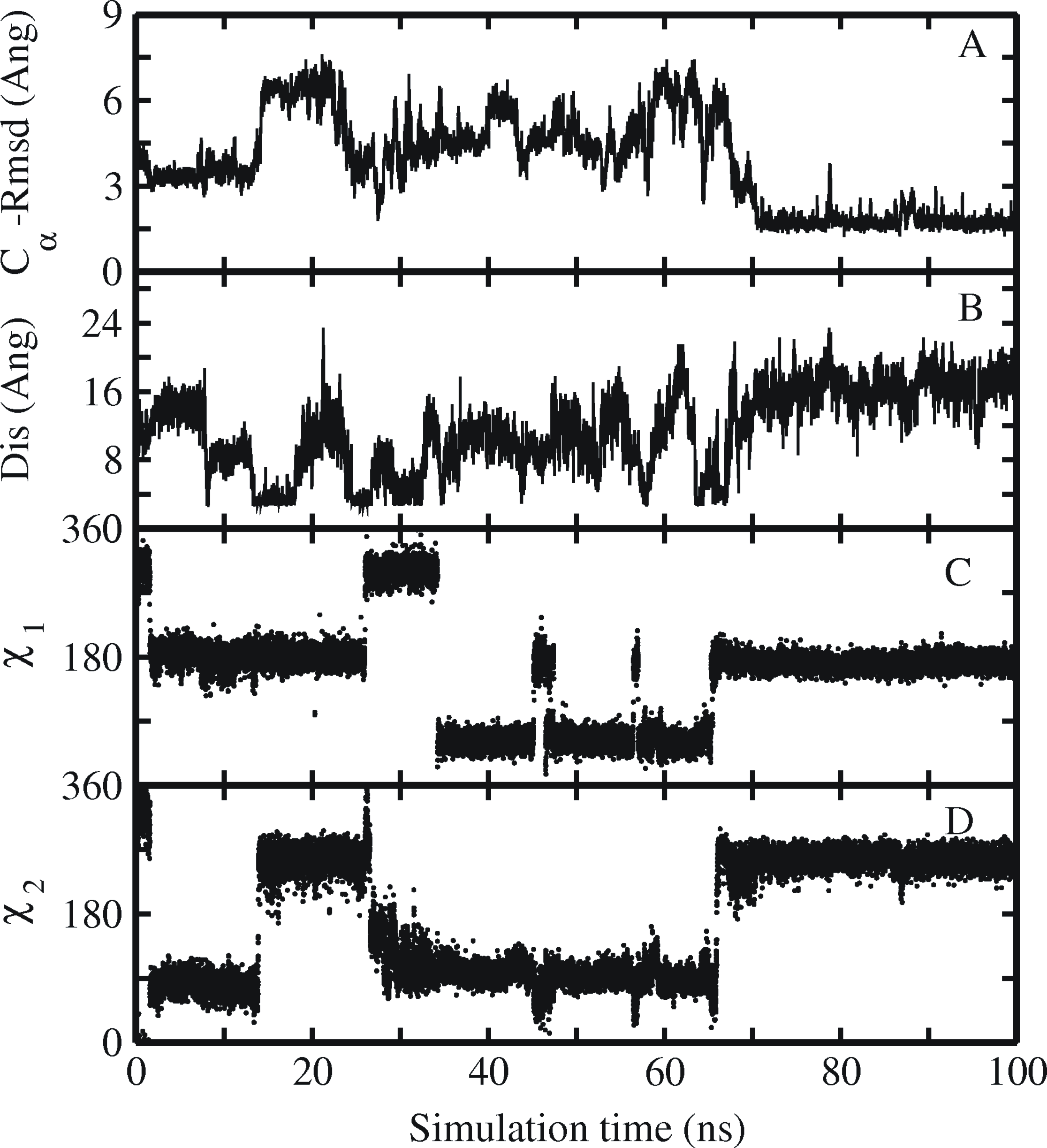

Supplement: Figure S6 — Time-dependence of backbone Rmsd and Trp6 side chain dihedral angles ff99SB_ILDN). (A) RMSDCα from native structure and (B) Asp9–Arg16 salt bridge distance as well as (C, D) side chain dihedral angels (χ1 and χ2) of Trp-6 residue of sampled Trp-cage conformations along one folding trajectory starting from a set2 intermediate structure vs. simulation time (force field ff99SB_ILDN). The dihedral angles of the native Trp6 side chain correspond to χ1 in trans (∼180°) and χ2 in –gauche (∼270°). (TIF) [file pone.0088383.s006.tif]

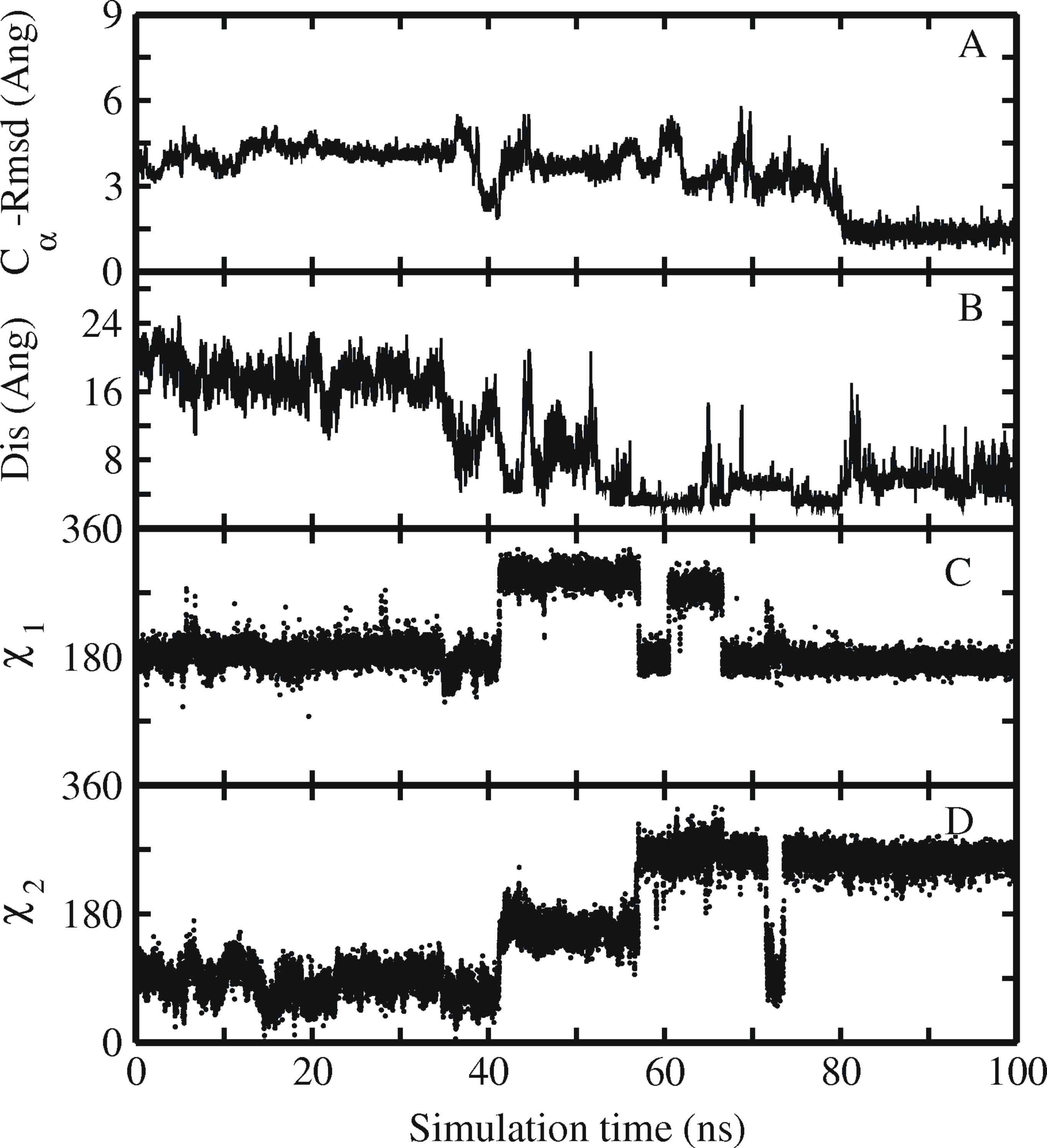

Supplement: Figure S7 — Time-dependence of backbone Rmsd and Trp6 side chain dihedral angles (ff99SB_NMR). (A) RMSDCα from native structure and (B) Asp9–Arg16 salt bridge distance as well as (C, D) side chain dihedral angels (χ1 and χ2) of Trp-6 residue of sampled Trp-cage conformations along one folding trajectory starting from a set2 intermediate structure vs. simulation time (force field ff99SB_NMR). The dihedral angles of the native Trp6 side chain correspond to χ1 in trans (∼180°) and χ2 in –gauche (∼270°). (TIF) [file pone.0088383.s007.tif]
